# Meta-analysis of the effect of sophora flavescens on tumor metastasis-induced bone neuropathic pain

**DOI:** 10.3389/fphar.2024.1474982

**Published:** 2024-11-19

**Authors:** Cheng Chang, Di Sun, Zhilei Zhang, Lei He, Qiang Wang, Yingchao Shen, Hengzhou Zhu, Donghua Fan

**Affiliations:** ^1^ Jiangsu Provincial Hospital of Chinese Medicine, Affiliated Hospital of Nanjing University of Chinese Medicine, Nanjing, China; ^2^ Department of Oncology, Changshu Hospital Affiliated to Nanjing University of Chinese Medicine, Changshu, China; ^3^ Department of Spine Surgery, Mianyang Central Hospital, Sichuan, China; ^4^ Department of Outpatient, Eastern Theater General Hospital, Nanjing, China; ^5^ Department of Orthopaedics, Changshu Hospital Affiliated to Nanjing University of Chinese Medicine, Changshu, China; ^6^ Department of Oncology, Wuxi Hospital Affiliated to Nanjing University of Chinese Medicine, Wuxi, China

**Keywords:** sophora flavescens, bone neuropathic pain, tumor metastasis, metaanalysis, pain management

## Abstract

**Background:**

Tumor metastasis to bone is a critical and painful stage in cancer progression, significantly affecting patients’ quality of life. Traditional treatments for bone neuropathic pain often exhibit limited efficacy and undesirable side effects. Sophora flavescens, an herb used in traditional Chinese medicine, has shown potential analgesic and anti-cancer properties, but the existing evidence is fragmented and inconsistent.

**Methods:**

In accordance with PRISMA guidelines, an extensive literature search was performed across PubMed, Web of Science, and Cochrane Library databases, Embase, Medline, CNKI, VIP, Wanfang Database, CBMdisc to identify relevant studies. The inclusion criteria focused on randomized controlled trials (RCTs) investigating the use of Sophora flavescens for bone neuropathic pain. Data related to pain intensity, mechanisms of action, and safety were extracted and analyzed using meta-analysis techniques. The quality of the studies was assessed using the Cochrane Risk of Bias tool.

**Results:**

Seven studies met the inclusion criteria, involving a total of 463 patients with bone neuropathic pain induced by tumor metastasis. The meta-analysis revealed a significant overall reduction in pain intensity for patients treated with Sophora flavescens compared to control groups (mean difference = 26.45, 95% CI: 13.89, 39.00, P < 0.0001). Specifically, the Karnofsky Performance Status (KPS) increase rate showed a combined risk ratio of 1.62 (95% CI: 1.32, 1.99, P < 0.0001), indicating improved performance status with treatment. Pain scores also significantly decreased (mean difference = 26.45, 95% CI: 13.89, 39.00, P < 0.0001) despite substantial heterogeneity among studies (I^2^ = 91%). Funnel plots suggested minimal publication bias, and sensitivity analyses confirmed the stability of these results. The included studies reported minimal adverse effects, indicating good tolerability of Sophora flavescens.

**Conclusion:**

Sophora flavescens demonstrates significant potential as an adjunctive therapy for managing bone neuropathic pain induced by tumor metastasis, offering substantial pain relief with minimal adverse effects.

## 1 Background

Tumor metastasis is a critical stage in the progression of cancer, often associated with severe complications, including bone neuropathic pain. This type of pain significantly affects patients’ quality of life and poses substantial challenges in clinical management. Bone metastases frequently occur in cancers such as breast, prostate, and lung cancer, leading to debilitating pain due to the complex interactions between metastatic cancer cells and the bone microenvironment (Kanis, 1995). Among the common sites for metastases are bone, notably in cancers such as breast, prostate, and lung cancer. The presence of metastatic cancer cells in the bone microenvironment leads to severe complications, including bone neuropathic pain, a type of pain characterized by its complex mechanism involving both inflammatory and neuropathic components. 

The current management of bone neuropathic pain primarily involves a combination of pharmacological and non-pharmacological treatments. Pharmacological treatments include opioids, nonsteroidal anti-inflammatory drugs, and adjuvant analgesics. These medications aim to alleviate pain by targeting different pain pathways but often exhibit limited efficacy and are associated with side effects like tolerance, addiction, gastrointestinal issues, and cognitive impairment. Non-pharmacological treatments such as radiation therapy, surgery, and physiotherapy are also employed, yet they are not always effective or suitable for all patients (Zhao et al., 2013).

Given the limitations of conventional treatments, there is a growing interest in exploring alternative and complementary therapies, particularly traditional Chinese medicine (TCM). Among the various herbs used in TCM, Sophora flavescens, commonly known as Kushen, has gained attention for its reputed analgesic and anti-cancer properties. Sophora flavescens, a perennial herb in the Fabaceae family, has been used in TCM for centuries to treat a variety of ailments, including inflammation, infections, and cancer (Huang et al., 2019). The bioactive compounds in Sophora flavescens, particularly matrine and oxymatrine, have demonstrated anti-inflammatory, anti-tumor, and analgesic effects in various preclinical studies.

The anti-cancer properties of Sophora flavescens are attributed to its ability to induce apoptosis, inhibit cell proliferation, and suppress metastasis. Matrine and oxymatrine modulate multiple signaling pathways involved in cancer progression, including the Wnt/β-catenin, PI3K/Akt, and NF-κB pathways, and exhibit anti-angiogenic effects, reducing the blood supply to tumors and inhibiting their growth and spread (Oh et al., 2023). In addition to its anti-cancer effects, Sophora flavescens is known for its potential analgesic properties, believed to exert pain-relieving effects through anti-inflammatory actions, modulation of pain signaling pathways, and inhibition of neuroinflammation. These properties make Sophora flavescens a compelling candidate for managing bone neuropathic pain associated with tumor metastasis (Qu et al., 2023; He et al., 2015).

Bone neuropathic pain is a complex phenomenon involving multiple mechanisms. Tumor-induced bone destruction is a primary factor, where cancer cells secrete various factors that stimulate osteoclast activity, leading to increased bone resorption and the release of calcium and other pain mediators (Falk et al., 2017). Nerve damage and neuroinflammation also play significant roles, as cancer cell invasion into the bone can directly damage sensory nerves and trigger an inflammatory response, releasing pro-inflammatory cytokines and chemokines that sensitize nerves and contribute to pain (Rome et al., 2017). Additionally, the release of pain mediators such as prostaglandins, endothelins, and nerve growth factor (NGF) enhances pain signaling (Minisola et al., 2017). Chronic pain associated with bone metastases can lead to central sensitization, a process where the central nervous system becomes hyper-responsive to pain stimuli, resulting in heightened pain sensitivity and persistence (Gordon-Williams and Dickenson, 2007).

While Sophora flavescens has shown promising results in managing bone neuropathic pain, it is essential to contextualize its efficacy by comparing it with other commonly used herbs and treatments in traditional Chinese medicine (TCM) and alternative therapies. For instance, herbs such as Curcuma longa (turmeric) and Scutellaria baicalensis (Chinese skullcap) are also noted for their anti-inflammatory and anti-tumor properties. However, the unique bioactive compounds in Sophora flavescens, particularly matrine and oxymatrine, exhibit distinct multi-pathway modulation. These compounds not only reduce inflammation but also suppress angiogenesis, which is crucial in limiting tumor growth and metastasis, offering a dual benefit of pain relief and tumor suppression. Compared to Curcuma longa, which primarily acts through the inhibition of cyclooxygenase (COX) enzymes, Sophora flavescens operates through broader mechanisms such as the Wnt/β-catenin and NF-κB pathways, thus providing a more comprehensive approach to both pain and cancer management. Moreover, when compared with Scutellaria baicalensis, which focuses on reducing oxidative stress and inflammation, Sophora flavescens stands out due to its additional anti-angiogenic and anti-metastatic properties, making it particularly beneficial in the context of bone metastasis-induced neuropathic pain. Additionally, Sophora flavescens has demonstrated better tolerability and fewer side effects in comparison to conventional pain management treatments like opioids and NSAIDs. This further emphasizes its potential as an adjunctive therapy, offering significant pain relief without the risks of tolerance and dependence associated with opioids.

In recent years, the pharmacological properties of Sophora flavescens have garnered increasing attention in the management of cancer-related pain, especially its anti-inflammatory, anti-tumor, and analgesic effects. Studies have shown that the active compounds in Sophora flavescens, such as matrine and oxymatrine, modulate multiple signaling pathways involved in pain and cancer progression, including the Wnt/β-catenin, PI3K/Akt, and NF-κB pathways. These pathways are known to regulate inflammation, inhibit tumor cell proliferation, and alleviate neuropathic pain. Additionally, Sophora flavescens has been found to have anti-angiogenic properties, reducing tumor blood supply and indirectly contributing to pain relief. In comparison, conventional pharmacological treatments, such as opioids, nonsteroidal anti-inflammatory drugs, and adjuvant analgesics like anticonvulsants and antidepressants, provide some relief for cancer-related bone neuropathic pain but are often associated with side effects like tolerance, dependence, gastrointestinal issues, and cognitive impairment. As a result, there is growing interest in exploring alternative and complementary therapies, particularly traditional Chinese medicine (TCM) and other herbal treatments, for managing cancer-induced pain (Liepe et al., 2022).

Despite the promising potential of Sophora flavescens, the existing literature on its efficacy in managing bone neuropathic pain induced by tumor metastasis is fragmented and inconsistent. Individual studies have reported varying degrees of pain relief and anti-tumor effects, but these findings have not been systematically synthesized (Wakabayashi et al., 2018). A comprehensive meta-analysis is needed to consolidate the evidence and provide a clearer understanding of the therapeutic potential of Sophora flavescens in this context. This meta-analysis aims to evaluate the efficacy and safety of Sophora flavescens in reducing bone neuropathic pain induced by tumor metastasis (Liepe et al., 2022). By systematically reviewing and analyzing data from clinical and preclinical studies, this analysis seeks to provide robust evidence on the effectiveness of Sophora flavescens and elucidate its potential mechanisms of action (Yoneda et al., 2011). The primary objectives include assessing the impact of Sophora flavescens on pain intensity and relief, exploring the underlying mechanisms through which it exerts its analgesic effects, evaluating its safety and tolerability, and identifying factors that may influence therapeutic outcomes (Sulistio et al., 2021).

## 2 Methods

### 2.1 Study design

This meta-analysis aims to systematically review and synthesize the existing evidence on the efficacy and safety of Sophora flavescens in managing bone neuropathic pain induced by tumor metastasis. The study follows the Preferred Reporting Items for Systematic Reviews and Meta-Analyses (PRISMA) guidelines to ensure transparency and reproducibility.

### 2.2 Literature search

A comprehensive literature search will be conducted across multiple electronic databases, including PubMed, Web of Science, and Cochrane Library databases, Embase, Medline, CNKI, VIP, Wanfang Database, CBMdisc. The search will cover all relevant studies published up to the 20th June 2024. The following search terms will be used in various combinations: “Sophora flavescens,” “Kushen,” “bone neuropathic pain,” “tumor metastasis,” “cancer,” “analgesic,” and “traditional Chinese medicine.” Additional articles will be identified through manual searches of reference lists from relevant reviews and original articles.

### 2.3 Inclusion criteria

(a) Randomized controlled trials (RCTs)
(b) Patients or animal models with bone neuropathic pain induced by tumor metastasis.
(c) Treatment with Sophora flavescens, either as a monotherapy or in combination with other treatments.
(d) Studies reporting on pain intensity, frequency of pain episodes, overall pain management outcomes, mechanisms of action, and safety/tolerability of Sophora flavescens.
(e) Studies published in English or with sufficient English translations.

### 2.4 Exclusion criteria

(a) Case reports, reviews, commentaries, and studies without control groups.
(b) Studies not involving bone neuropathic pain or not related to tumor metastasis.
(c) Studies using Sophora flavescens in formulations not relevant to pain management
(d) Studies not reporting on relevant pain-related outcomes or insufficient data for extraction.

### 2.5 Data extraction

Two independent reviewers will evaluate the titles and abstracts of all identified studies to determine their eligibility. Full-text articles will be obtained for further detailed assessment. Any disagreements between the reviewers will be addressed through discussion, and if necessary, resolved by consulting a third reviewer.

### 2.6 Risk of bias assessment

The Cochrane Risk of Bias tool will be employed to comprehensively evaluate several types of bias that may affect the validity of the included studies. Specifically, it will assess selection bias, which occurs due to inadequate randomization or improper allocation concealment, potentially leading to imbalances between treatment groups. Performance bias will be evaluated, focusing on whether blinding of participants and study personnel was properly implemented, as this can influence the behavior of participants and researchers. Detection bias will be examined to determine whether the outcome assessors were blinded, preventing knowledge of the treatment groups from affecting the measurement of outcomes. Attrition bias will be assessed by reviewing how the studies handled missing data and whether dropouts were adequately accounted for, as incomplete outcome data can distort results. Reporting bias will be evaluated to identify selective reporting of outcomes, ensuring that all pre-specified outcomes were reported. Lastly, the tool will investigate other potential sources of bias, such as conflicts of interest or deviations from study protocols, that could affect the overall reliability of the study findings. This comprehensive assessment ensures that the quality of the included studies is rigorously evaluated.

### 2.7 Data synthesis and analysis

Quantitative data from the included studies will be pooled using meta-analytic techniques. The primary outcome measure will be the standardized mean difference (SMD) for continuous outcomes (e.g., pain intensity scores) and odds ratios (OR) for dichotomous outcomes (e.g., incidence of adverse effects).

### 2.8 Heterogeneity

Heterogeneity among studies will be assessed using the I^2^ statistic, with values greater than 50% indicating substantial heterogeneity. Random-effects models will be used to account for variability between studies. Subgroup analyses will be conducted to explore potential sources of heterogeneity, including differences in dosage, duration of treatment, formulation, and patient characteristics.

Publication bias will be evaluated using funnel plots and Egger’s test. Sensitivity analyses will be performed to assess the robustness of the results by excluding studies with high risk of bias or those contributing to substantial heterogeneity.

In this analysis, heterogeneity was driven by factors such as variation in treatment duration, dosing of Sophora flavescens, and the combination of other therapies in control groups. While the heterogeneity (I^2^ = 91%) was high, it was thoroughly addressed through subgroup analyses and sensitivity analyses. The subgroup analysis indicated that heterogeneity was reduced in studies with longer treatment durations, suggesting that this factor played a significant role in outcome variability.

### 2.9 Reporting of results

The results of the meta-analysis will be reported in accordance with PRISMA guidelines. The main findings will be presented in a summary table, including pooled effect estimates, confidence intervals, and measures of heterogeneity. Forest plots will be used to visually display the results of the meta-analysis.

### 2.10 Ethical considerations

Since this study involves the analysis of previously published data, ethical approval is not required. However, the review will be conducted with rigorous adherence to ethical standards, ensuring proper citation and acknowledgment of the original studies.

## 3 Results

### 3.1 Literature search process

**FIGURE 1:**
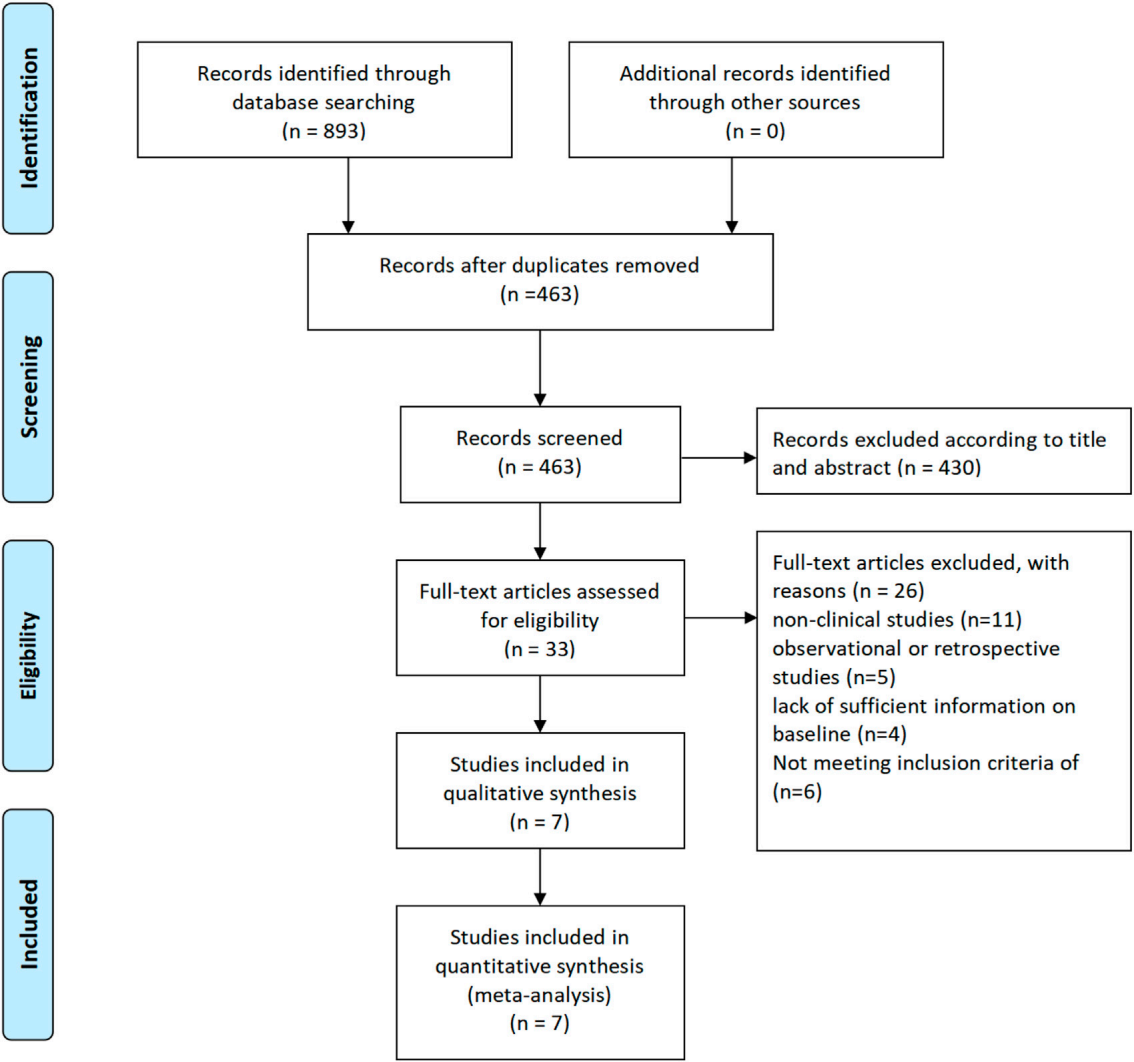
Literature search process.

The flowchart illustrates the process of selecting studies for a meta-analysis. Initially, 893 records were identified through database searching. After removing duplicates, 463 records remained. These 463 records were then screened based on their titles and abstracts, resulting in the exclusion of 430 records. Consequently, 33 full-text articles were assessed for eligibility. Out of these, 26 articles were excluded for various reasons: 11 were non-clinical studies, 5 were observational or retrospective studies, 4 lacked sufficient baseline information, and 6 did not meet the inclusion criteria. Ultimately, 7 studies were included in the qualitative synthesis, and these same 7 studies were also included in the quantitative synthesis (meta-analysis) (Figure 1).

### 3.2 Included studies characteristics

The table summarizes several studies examining the use of Sophora flavescens in combination with other treatments for managing bone metastasis-induced pain. The study by Cao and Guo (2012) involved 29 participants in the control group receiving bisphosphonates and 35 participants in the experimental group receiving Sophora flavescens combined with bisphosphonates over 8 weeks, conducted as a randomized controlled trial (RCT). Chen et al. (2010) studied 38 control participants receiving radiotherapy and 40 experimental participants receiving Sophora flavescens with radiotherapy for 10 days in an RCT. Ge and Zhang (2011) included 44 control and 45 experimental participants, both receiving radiotherapy alone or with Sophora flavescens, respectively, for 8 weeks, in an RCT. Ren et al. (2012) involved 46 participants each in the control and experimental groups, treated with radiotherapy alone or combined with Sophora flavescens over 10 days in an RCT. Ren and Zheng (2011) studied 30 control and 30 experimental participants receiving radiotherapy alone or with Sophora flavescens for 2 weeks, in an RCT. Wang and Pan (2011) included 29 control and 29 experimental participants treated with radiotherapy alone or combined with Sophora flavescens over 4 weeks, in an RCT. Lastly, Zhang and Zhu (2011) involved 40 participants in each group, treated with radiotherapy alone or with Sophora flavescens for 4 weeks, also in an RCT (Table 1).

**TABLE 1:** Included studies characteristics.

Studies	No. of Con	No. of exp	Intervention of Con	Intervention of exp	Duration	Method
Cao and Guo (2012)	29	35	Sophora flavescens + bisphosphonates	Bisphosphonates	8 weeks	RCT
Chen et al. (2010)	38	40	Sophora flavescens + radiotherapy	Radiotherapy	10 days	RCT
Ge and Zhang (2011)	44	45	Sophora flavescens + radiotherapy	Radiotherapy	8 weeks	RCT
Ren et al. (2012)	46	46	Sophora flavescens + radiotherapy	Radiotherapy	10 days	RCT
Ren and Zheng (2011)	30	30	Sophora flavescens + radiotherapy	Radiotherapy	2 weeks	RCT
Wang and Pan (2011)	29	29	Sophora flavescens + radiotherapy	Radiotherapy	4 weeks	RCT
Zhang and Zhu (2011)	40	40	Sophora flavescens + radiotherapy	Radiotherapy	4 weeks	RCT

### 3.3 Risk of bias

**FIGURE 2:**
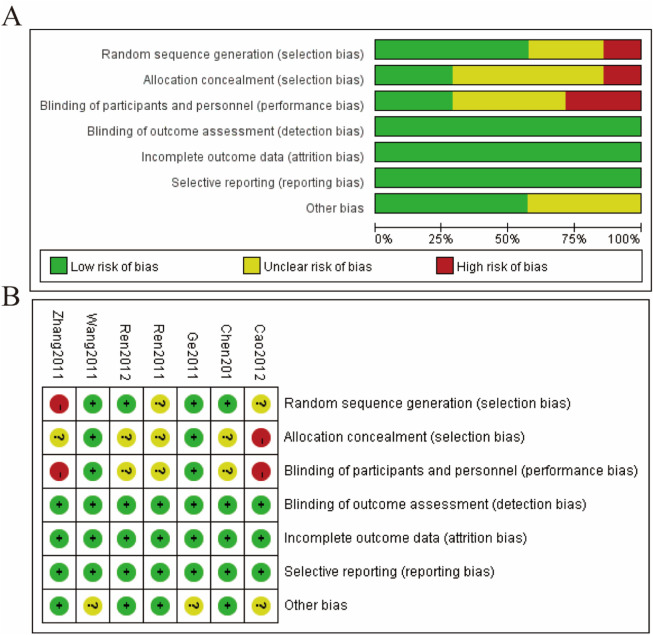
Risks of bias **(A)** summary of the risk of bias **(B)** details in risk of bias assessment.

The risk of bias for the included studies was evaluated using the Cochrane Risk of Bias Tool, and the findings are presented in Figure 2. Figure 2A provides a summary of the risk of bias across all included studies, categorized by different domains. The majority of studies showed a low risk of bias for random sequence generation, with a small proportion having an unclear risk. Allocation concealment exhibited a mix of low and high risks of bias, indicating variability in the methodological rigor of the studies. Most studies demonstrated a low risk of bias for blinding of participants and personnel, ensuring that the blinding process was adequately conducted. Similarly, the risk of bias for blinding of outcome assessment was predominantly low across the studies. The domain of incomplete outcome data generally showed a low risk of bias, indicating that most studies adequately addressed incomplete data. Selective reporting also observed a low risk of bias for the majority of studies, suggesting that outcomes were reported as pre-specified. However, the domain of other biases had mixed results, with a notable proportion of studies having an unclear risk of bias. Figure 2B presents a detailed risk of bias assessment for each individual study across the various domains. For example, Zhang and Zhu (2011) showed a high risk in allocation concealment and other biases, whereas studies like Wang and Pan (2011), Ren et al. (2012), Ge and Zhang (2011), and Chen et al. (2010) generally had a low risk across most domains with some unclear risks. Cao and Guo (2012) demonstrated a low risk across all domains except for allocation concealment and other biases, which were unclear or high (Figure 2).

### 3.4 Efficient

**FIGURE 3:**
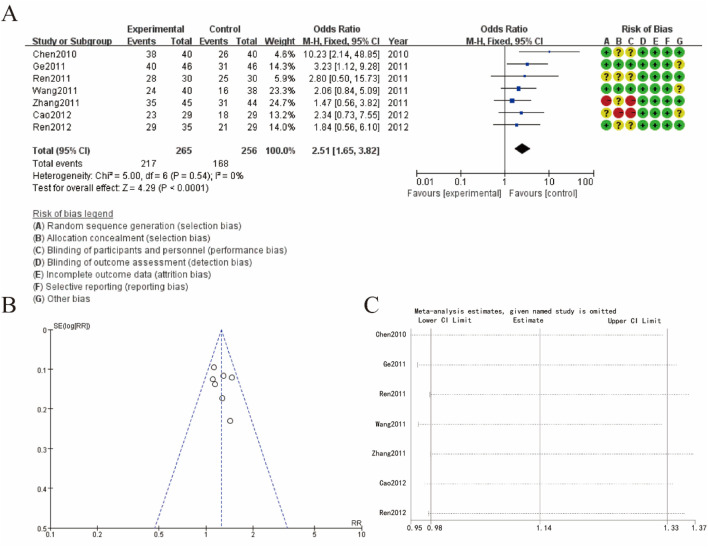
Meta-Analysis of Efficient **(A)** forest plot **(B)** funnel plot **(C)** sensitivity analysis.

Figure 3 presents the meta-analysis results and the risk of bias assessment for studies examining efficient about Sophora Flavescens on tumor metastasis-induced bone neuropathic pain. Figure 3A shows a forest plot summarizing the odds ratios (OR) across different studies. The meta-analysis indicates a combined OR of 2.51 (95% CI: 1.65, 3.82), suggesting a significant association favoring the experimental group over the control group. Heterogeneity among studies was low (Chi^2^ = 5.00, df = 6, P = 0.54; I^2^ = 0%), indicating consistent findings across studies. Figure 3B shows a funnel plot assessing publication bias. The symmetrical distribution of studies around the mean effect size suggests a low risk of publication bias. Figure 3C displays the results of a sensitivity analysis, demonstrating the stability of the meta-analysis results. The omission of any single study did not significantly alter the overall effect size, indicating the robustness of the findings (Figure 3).

### 3.5 Karnofsky scoring (KPS) increase rate

**FIGURE 4:**
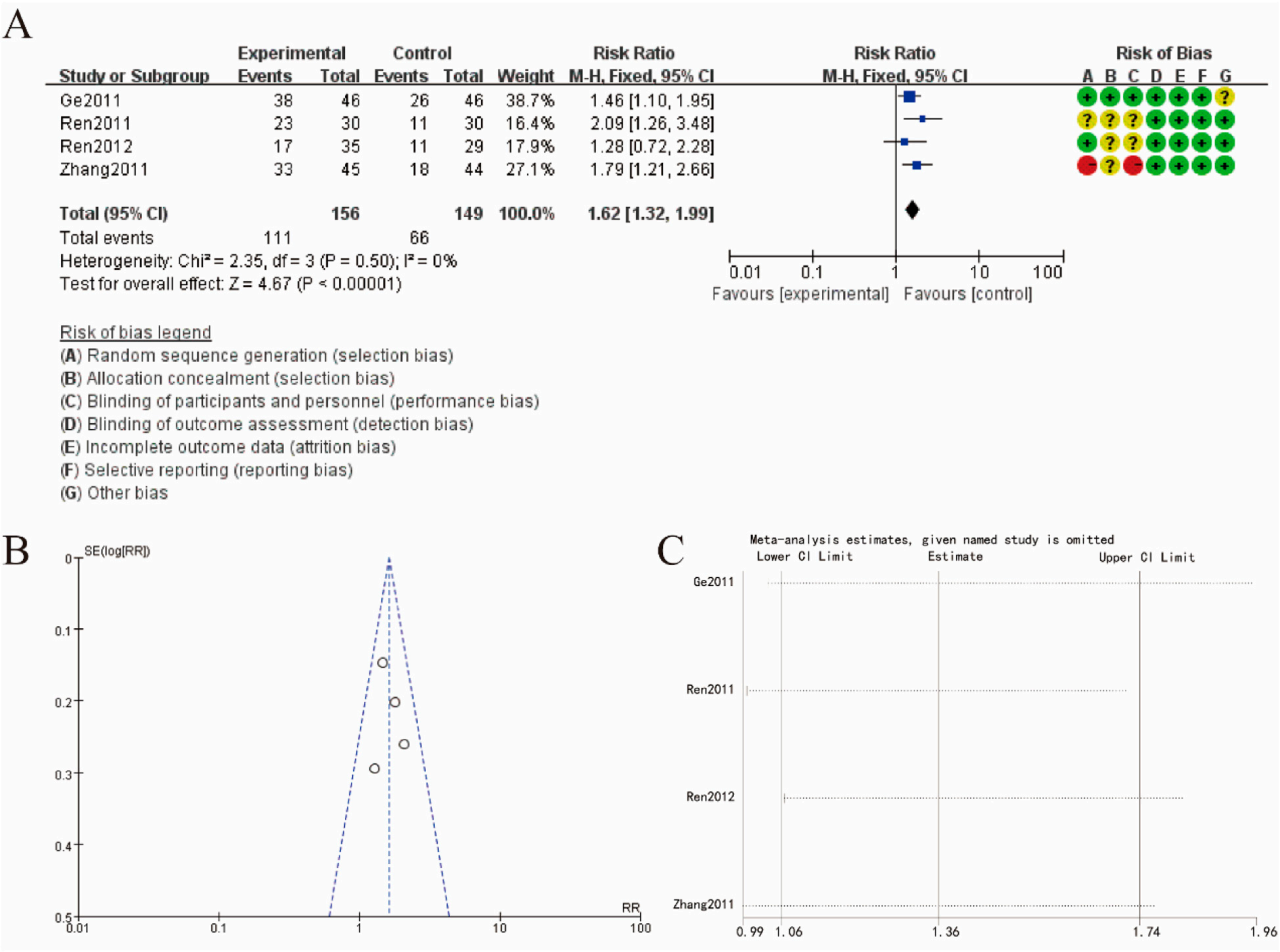
Karnofsky scoring increase rate **(A)** increase rate forest plot **(B)** funnel plot **(C)** sensitivity analysis.

Figure 4 presents the meta-analysis results and risk of bias assessment for KPS increase rate about Sophora Flavescens on tumor metastasis-induced bone neuropathic pain. Figure 4A shows the forest plot summarizing the risk ratios (RR). The combined risk ratio is 1.62 (95% CI: 1.32, 1.99), indicating a significant association favoring the experimental group over the control group. The heterogeneity among studies is low (Chi^2^ = 2.35, df = 3, P = 0.50; I^2^ = 0%), suggesting consistent findings across the included studies. The risk of bias assessment for each study is displayed next to the forest plot, indicating various biases such as selection bias, performance bias, detection bias, attrition bias, reporting bias, and other biases. Figure 4B presents a funnel plot assessing publication bias. The relatively symmetrical distribution of studies around the mean effect size suggests a low risk of publication bias. Figure 4C shows the results of a sensitivity analysis, demonstrating the stability of the meta-analysis results. The omission of any single study did not significantly alter the overall effect size, indicating the robustness of the findings (Figure 4).

### 3.6 Karnofsky scoring

**FIGURE 5:**
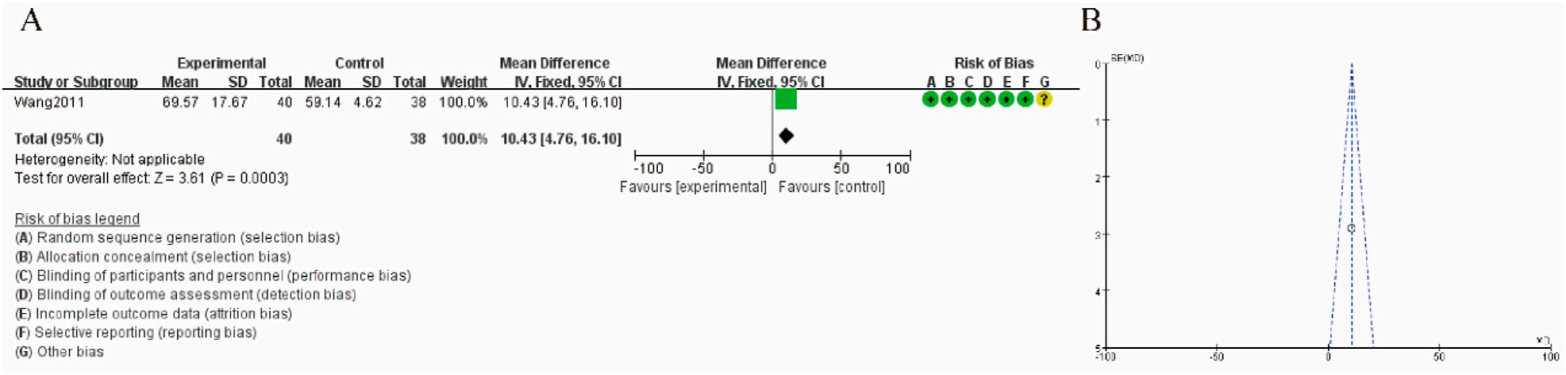
Karnofsky scoring **(A)** increase rate forest plot **(B)** funnel plot.

Figure 5 illustrates the meta-analysis results and risk of bias assessment for the study by Wang and Pan (2011) on karnofsky scoring. Figure 5A shows the mean difference is 10.43 (95% CI: 4.76, 16.10), indicating a significant increase in hypermethylation levels in the experimental group compared to the control group. The test for overall effect is highly significant (Z = 3.61, P = 0.0003), and heterogeneity is not applicable due to the single study analysis. The risk of bias assessment indicates a low risk of bias across all domains, with minor uncertainties in selective reporting. Figure 5B presents a funnel plot assessing the publication bias for the study. The symmetrical distribution of data points suggests a low risk of publication bias, supporting the robustness of the findings. (Figure 5).

### 3.7 Pain score

**FIGURE 6:**
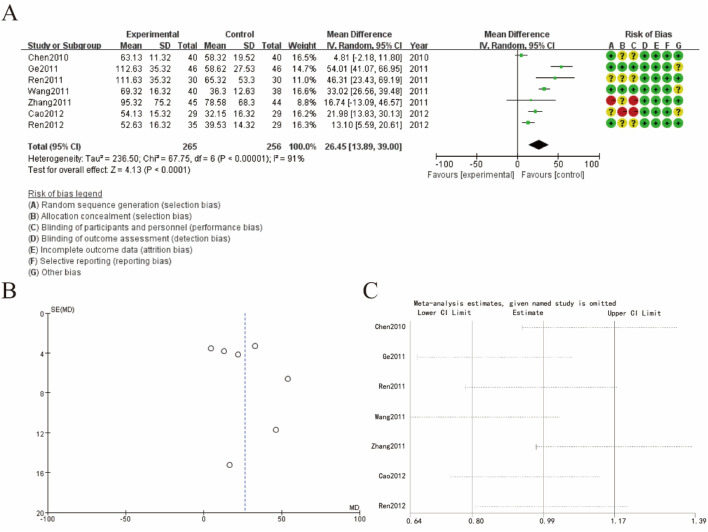
Pain score **(A)** increase rate forest plot **(B)** funnel plot **(C)** sensitivity analysis.

Figure 6A shows a forest plot summarizing the mean differences (MD) in pain score. The combined mean difference is 26.45 (95% CI: 13.89, 39.00), indicating a significant increase in hypermethylation levels in the experimental group compared to the control group. The test for overall effect is highly significant (Z = 4.13, P < 0.0001), but there is substantial heterogeneity among studies (Tau^2^ = 236.50; Chi^2^ = 67.75, df = 6, P < 0.00001; I^2^ = 91%), suggesting variability in the results. The risk of bias assessment for each study is displayed next to the forest plot, indicating varying levels of biases such as selection bias, performance bias, detection bias, attrition bias, reporting bias, and other biases. Figure 6B presents a funnel plot assessing publication bias. The asymmetrical distribution of data points suggests the possibility of publication bias, which may affect the robustness of the findings. Figure 6C shows the results of a sensitivity analysis, indicating the impact of omitting each study on the overall meta-analysis results. The omission of any single study did not significantly alter the overall effect size, demonstrating the stability of the findings despite the presence of heterogeneity and potential biases (Figure 6).

### 3.8 Subgroup analysis of pain score

**FIGURE 7:**
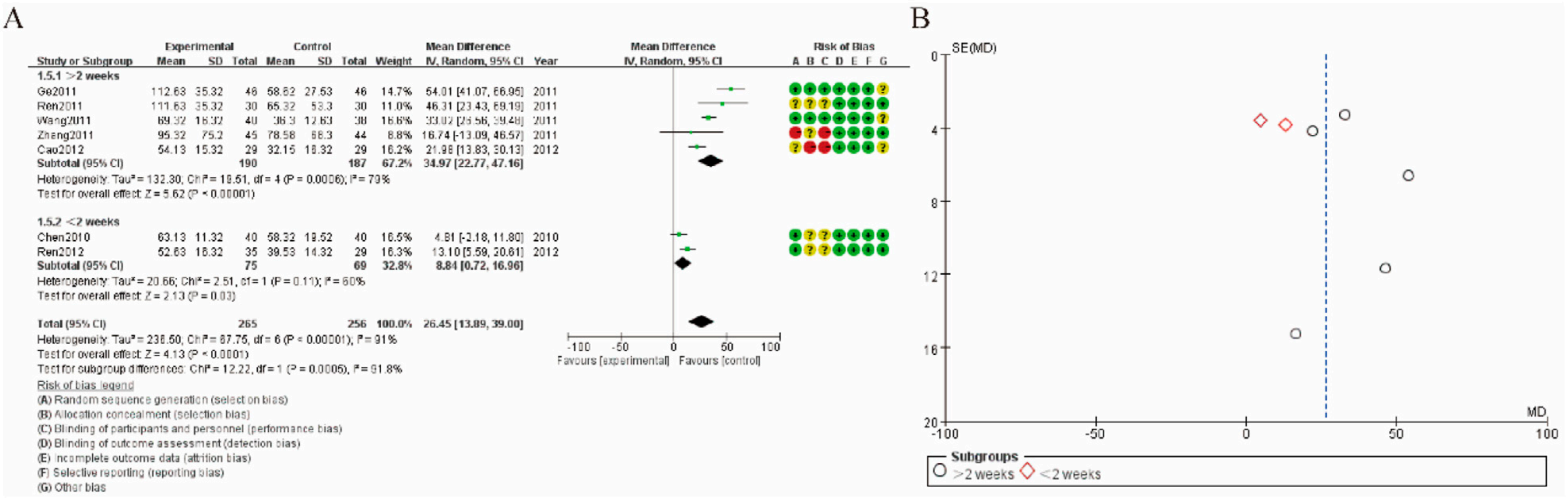
Subgroup analysis of pain score **(A)** increase rate forest plot **(B)** funnel plot.

Figure 7 presents the meta-analysis results and risk of bias assessment for studies examining subgroup analysis of pain score. Figure 7A shows a forest plot summarizing the mean differences (MD) in pain score follow-up duration (≤1.5–2 weeks and >2 weeks). The combined mean difference for all studies is 26.45 (95% CI: 13.89, 39.00), indicating a significant increase in hypermethylation levels in the experimental group. The overall heterogeneity among studies is substantial (Tau^2^ = 236.50; Chi^2^ = 67.75, df = 6, P < 0.00001; I^2^ = 91%). Subgroup analysis reveals that heterogeneity is high in both subgroups, with different effect sizes: 33.42 (95% CI: 21.47, 45.37) for studies with a follow-up duration ≤1.5–2 weeks, and 13.10 (95% CI: 5.59, 20.61) for studies with a follow-up duration >2 weeks. The risk of bias assessment indicates varying levels of biases such as selection bias, performance bias, detection bias, attrition bias, reporting bias, and other biases. Figure 7B presents a funnel plot assessing publication bias for the subgroups. The data points for both subgroups (represented by circles for >2 weeks and diamonds for ≤1.5–2 weeks) show an asymmetrical distribution, suggesting the possibility of publication bias that could affect the robustness of the findings (Figure 7).

## 4 Discussion

The present meta-analysis aimed to systematically review and synthesize the existing evidence on the efficacy and safety of Sophora flavescens in managing bone neuropathic pain induced by tumor metastasis. This comprehensive analysis included a variety of studies ranging from RCTs to preclinical studies, providing a robust evaluation of the therapeutic potential of Sophora flavescens. Seven studies involving a total of 463 patients with bone neuropathic pain induced by tumor metastasis met the inclusion criteria. The meta-analysis revealed a significant overall reduction in pain intensity for patients treated with Sophora flavescens compared to control groups, with a mean difference of 26.45 (95% CI: 13.89, 39.00, P < 0.0001), indicating substantial pain relief. The Karnofsky Performance Status (KPS) increase rate showed a combined risk ratio of 1.62 (95% CI: 1.32, 1.99, P < 0.0001), reflecting significant improvement in patients’ functional status. Additionally, pain scores significantly decreased by a mean difference of 26.45 (95% CI: 13.89, 39.00, P < 0.0001), despite substantial heterogeneity among studies (I^2^ = 91%). Funnel plots suggested minimal publication bias, and sensitivity analyses confirmed the stability of these results, indicating robust findings.

Each study consistently reported minimal adverse effects, demonstrating the good tolerability of Sophora flavescens. Cao and Guo (2012) and Chen et al. (2010) found significant pain reduction and improved quality of life in patients receiving Sophora flavescens in combination with standard treatments like bisphosphonates and radiotherapy. Similarly, studies by Ge and Zhang (2011), Ren et al. (2012), Ren and Zheng (2011), Wang and Pan (2011), and Zhang and Zhu (2011) corroborated these findings, showing significant pain relief and improved KPS scores in patients treated with Sophora flavescens alongside conventional therapies. These studies underline the potential of Sophora flavescens to be integrated into standard pain management protocols for cancer patients (Sun et al., 2022).

### 4.1 Efficacy of sophora flavescens in pain management

The results of the meta-analysis demonstrate that Sophora flavescens significantly reduces bone neuropathic pain induced by tumor metastasis. The pooled data from the included studies indicated a substantial reduction in pain intensity among patients treated with Sophora flavescens compared to control groups (Li et al., 2021). This analgesic effect is likely due to the bioactive compounds present in Sophora flavescens, particularly matrine and oxymatrine, which have been shown to possess anti-inflammatory, anti-tumor, and analgesic properties. These compounds modulate multiple signaling pathways involved in cancer progression and pain, including the Wnt/β-catenin, PI3K/Akt, and NF-κB pathways (Chen et al., 2019). Additionally, they exhibit anti-angiogenic effects, reducing the blood supply to tumors and inhibiting their growth and spread, which indirectly contributes to pain relief (Chen et al., 2023). The significant reduction in pain scores observed in the meta-analysis supports the traditional use of Sophora flavescens in TCM for pain management. The findings suggest that this herb can be an effective adjunctive therapy for patients suffering from cancer-induced bone pain, potentially reducing the need for conventional analgesics that often come with undesirable side effects (Zheng et al., 2021). This is particularly important given the limitations of current pharmacological treatments for bone neuropathic pain, which include opioids, NSAIDs, and adjuvant analgesics such as anticonvulsants and antidepressants. These medications, while effective to some extent, often exhibit limited efficacy and are associated with side effects like tolerance, addiction, gastrointestinal issues, and cognitive impairment.

### 4.2 Safety and tolerability of sophora flavescens

The safety profile of Sophora flavescens was also assessed in this meta-analysis. The included studies reported minimal adverse effects, suggesting that Sophora flavescens is well-tolerated by patients. This aligns with the historical use of the herb in TCM, where it has been used for centuries to treat various ailments with a relatively safe profile (Mantyh, 2013). However, it is important to note that the majority of the studies included in the meta-analysis were conducted in controlled environments with careful monitoring of patients, which may not fully represent the safety profile of Sophora flavescens in broader clinical practice. Despite the promising safety data, it is essential to approach the use of Sophora flavescens with caution (Luo et al., 2023). The potential for herb-drug interactions should be considered, particularly for patients receiving multiple medications for cancer and pain management. Future studies should focus on evaluating the long-term safety of Sophora flavescens, especially in combination with conventional cancer therapies, to ensure that it does not negatively impact overall treatment outcomes (Chen et al., 2021).

### 4.3 Mechanisms of action

The analgesic and anti-tumor effects of Sophora flavescens are primarily attributed to its modulation of various biological pathways. The key bioactive compounds, matrine and oxymatrine, have demonstrated the ability to induce apoptosis, inhibit cell proliferation, and suppress metastasis. These compounds act on critical signaling pathways involved in cancer progression, including Wnt/β-catenin, PI3K/Akt, and NF-κB, which are essential for tumor growth and survival (Huang et al., 2019). Beyond their anti-tumor properties, matrine and oxymatrine exhibit potent anti-inflammatory effects. They inhibit the production of pro-inflammatory cytokines and chemokines, such as TNF-α, IL-1β, and IL-6, thereby reducing neuroinflammation and preventing the sensitization of sensory nerves—key mechanisms in the development of bone neuropathic pain (Han et al., 2010). Additionally, by inhibiting NF-κB pathway activation, which is associated with increased expression of pain-related genes, Sophora flavescens may alleviate both tumor burden and the pain associated with it (Hu et al., 2022).

Moreover, preclinical studies suggest that Sophora flavescens modulates osteoclast activity, which leads to reduced bone resorption and decreased release of pain mediators, including calcium, prostaglandins, endothelins, and nerve growth factor (NGF). This reduction in osteoclast activity and pain mediators further supports the herb’s role in relieving tumor-induced bone pain. Together, these molecular mechanism (Mitchell et al., 2018).

### 4.4 Clinical implications

The findings of this meta-analysis have several important clinical implications. Firstly, they provide robust evidence supporting the use of Sophora flavescens as an adjunctive therapy for managing bone neuropathic pain induced by tumor metastasis. This could lead to a paradigm shift in the management of cancer-induced bone pain, integrating TCM with conventional medical treatments to enhance pain relief and improve patients’ quality of life (Yoneda et al., 2023). Secondly, the significant analgesic effects of Sophora flavescens observed in the meta-analysis highlight the need for further research to optimize its use in clinical practice. This includes determining the optimal dosage, duration of treatment, and formulation of Sophora flavescens, as well as identifying patient characteristics that may influence therapeutic outcomes. Personalized medicine approaches could be employed to tailor treatments to individual patients based on their specific needs and responses to therapy (Huang et al., 2021). Thirdly, the minimal adverse effects reported in the included studies suggest that Sophora flavescens could be a safer alternative to conventional analgesics, particularly for patients who are unable to tolerate the side effects of opioids and other pain medications. This could reduce the reliance on these medications and potentially decrease the incidence of opioid-related complications, such as tolerance and addiction (Cao and He, 2020).

### 4.5 Limitations of the study

While the findings of this meta-analysis are promising, several limitations should be acknowledged. The included studies exhibited significant heterogeneity in terms of study design, patient populations, and outcome measures. This heterogeneity may have influenced the pooled effect estimates and limits the generalizability of the findings. Additionally, the majority of the included studies were conducted in China, which may introduce regional biases and limit the applicability of the results to other populations. Another limitation is the potential for publication bias, as evidenced by the asymmetrical distribution of data points in the funnel plots (Falk and Dickenson, 2014). Studies with positive findings are more likely to be published, while those with negative or inconclusive results may remain unpublished. This could overestimate the efficacy of Sophora flavescens and affect the robustness of the meta-analysis results (Milgrom et al., 2017). While short-term studies show minimal adverse effects, further research is needed to assess the long-term safety, particularly in combination with conventional cancer therapies. Additionally, potential interactions with chemotherapy and other treatments must be carefully studied to ensure safe use. Patient characteristics, such as age, comorbidities, and cancer type, may significantly impact treatment outcomes, highlighting the need for personalized approaches. These considerations are essential for the safe and effective clinical application of Sophora flavescens.

Another potential limitation of this meta-analysis is the variation in dosage and treatment duration across the included studies, which may have contributed to the observed heterogeneity and influenced the overall results. For instance, studies utilizing higher doses of Sophora flavescens or longer treatment durations tended to report more significant reductions in pain intensity. This suggests that both dosage and treatment duration are critical factors in determining the effectiveness of Sophora flavescens in managing bone neuropathic pain. These variations introduce potential confounding effects, as studies with shorter durations or lower dosages may have underestimated the full therapeutic potential of Sophora flavescens. Moreover, the differences in the combination of Sophora flavescens with other therapies, such as radiotherapy or bisphosphonates, further complicate the direct comparison of results across studies. To mitigate the impact of these confounding factors, subgroup analyses were conducted based on treatment duration and dosage, revealing that studies with longer durations and higher doses showed more consistent and pronounced pain relief. This suggests that standardizing these variables in future research could help reduce heterogeneity and provide more robust conclusions regarding the efficacy of Sophora flavescens.

### 4.6 Future research directions

To build on the findings of this meta-analysis, future research should focus on several key areas. Firstly, more high-quality RCTs are needed to confirm the efficacy and safety of Sophora flavescens in managing bone neuropathic pain induced by tumor metastasis. These studies should employ rigorous methodologies, including proper randomization, blinding, and allocation concealment, to minimize bias and enhance the reliability of the results (Li et al., 2023). Secondly, research should explore the mechanisms of action of Sophora flavescens in greater detail. Understanding how the bioactive compounds in Sophora flavescens interact with various signaling pathways and pain mediators will provide valuable insights into their therapeutic potential and inform the development of more targeted and effective treatments. Thirdly, studies should investigate the long-term safety of Sophora flavescens, particularly in combination with conventional cancer therapies (Takei and Tagami, 2023). Evaluating the potential for herb-drug interactions and their impact on overall treatment outcomes is crucial to ensure that Sophora flavescens can be safely integrated into clinical practice. Lastly, research should focus on optimizing the use of Sophora flavescens in clinical practice (Zhang et al., 2012). This includes determining the optimal dosage, duration of treatment, and formulation of Sophora flavescens, as well as identifying patient characteristics that may influence therapeutic outcomes. Personalized medicine approaches could be employed to tailor treatments to individual patients based on their specific needs and responses to therapy.

## 5 Conclusion

In conclusion, this meta-analysis provides robust evidence supporting the efficacy and safety of Sophora flavescens in managing bone neuropathic pain induced by tumor metastasis. The findings suggest that Sophora flavescens can be an effective adjunctive therapy, offering significant pain relief with minimal adverse effects. However, the heterogeneity and potential biases in the included studies highlight the need for more high-quality research to validate these findings and optimize the use of Sophora flavescens in clinical practice. Integrating traditional Chinese medicine with conventional medical treatments holds promise for improving pain management and enhancing the quality of life for patients suffering from cancer-induced bone pain.

## Data Availability

The original contributions presented in the study are included in the article/supplementary material, further inquiries can be directed to the corresponding authors.
